# Health Belief Model Predicts Likelihood of Eating Nutrient-Rich Foods among U.S. Adults

**DOI:** 10.3390/nu16142335

**Published:** 2024-07-19

**Authors:** Abigail A. Glick, Donna M. Winham, Michelle M. Heer, Mack C. Shelley, Andrea M. Hutchins

**Affiliations:** 1 Department of Food Science & Human Nutrition, Iowa State University, Ames, IA 50011, USA; aaglick@iastate.edu (A.A.G.); mmheer@iastate.edu (M.M.H.); 2 Departments of Political Science and Statistics, Iowa State University, Ames, IA 50011, USA; mshelley@iastate.edu; 3 Department of Human Physiology & Nutrition, University of Colorado Colorado Springs, Colorado Springs, CO 80918, USA; ahutchin@uccs.edu

**Keywords:** nutrients, chronic disease, nutrition knowledge, under-consumed nutrients, nutrient density, structural equation model, nutrient rich foods, health belief model, consumer research

## Abstract

Despite decades of messaging, most Americans still consume excess fats and sugars, but inadequate fiber, potassium, and calcium. Nutrient-rich foods (NRFs) have a high density of favorable nutrients related to calories. Choosing NRFs could lower risk of nutrition-related chronic diseases and aid in their control. We hypothesized that having greater knowledge of NRFs, the presence of a nutrition-related chronic disease or risk factor, and positive Health Belief Model (HBM) views would be predictive of the likelihood of eating NRFs. Through a national online survey panel, 976 adults aged 18–80 completed demographic, health, NRF knowledge, attitudes, and HBM construct questions. Participants were 77% White, 52% women, and 55% had a nutrition-related disease or risk factor. Multivariable HBM scales were generated by theory, principal components, and reliability analysis. NRF knowledge was significantly higher for women, Whites, households without children, and persons without a nutrition-related disease (all *p* ≤ 0.015). ‘Likelihood of eating NRFs’ was significantly higher for persons with a nutrition-related disease, Whites, married participants, main food shoppers, and households with children (all *p* ≤ 0.022). Regressing demographic and HBM constructs on the ‘likelihood of eating NRFs’ resulted in *R*^2^ of 0.435. Nutrition-related disease and HBM constructs of self-efficacy, perceived benefits, and cues to action were predictive of the likelihood of eating NRFs, but higher NRF knowledge was negatively associated.

## 1. Introduction

Nutrition-related conditions like high blood pressure, elevated cholesterol, cardiovascular disease, and type 2 diabetes remain risk factors for morbidity and mortality for Americans [1]. While the United States (U.S.) Dietary Guidelines for Americans (DGA) have encouraged consumption or limitation of certain foods to reduce chronic disease susceptibility for decades, the majority of Americans do not meet these recommendations [2]. The DGA have identified five shortfall nutrients for adults: dietary fiber, calcium, potassium, vitamin D, and iron for women of childbearing age and their infants [2]. Beyond shortfall nutrients, the DGA recommends intakes of macronutrients which can aid in the management and prevention of chronic disease, such as lower carbohydrate, saturated fat, and sodium diets when appropriate, and higher-protein diets to support lean body mass [3].

Nutrient-dense or nutrient-rich foods (NRFs) contain higher levels of fiber, vitamins, minerals, and protein with less sodium, added sugar, and saturated fat [4]. Ample evidence supports the positive effects of NRFs on health, which is in line with preventative medicine efforts [5,6]. Excessive sodium in the American diet can elevate blood pressure and cardiovascular disease [2]. An increase in simple carbohydrates, specifically added sugars, can raise the risk of diabetes, cardiovascular disease, and obesity [7]. Similarly, dietary saturated fat intakes have been associated with greater cardiovascular disease, and weight gain due to their higher number of calories per gram [2,6,8]. Consumption of NRFs is a feasible way to decrease the risk for nutrient deficiencies and to consume foods lower in added sugar, saturated fat, and sodium [4,8]. However, in a 2019 national survey, only 18% of Americans felt ‘very confident’ in their ability to identify nutrient-dense foods [9]. 

Although the DGA are known among the professional nutrition community, the public knowledge of the DGA tends not to meet the threshold to promote behavior change [2,10,11]. Barriers to following the DGA, or healthy eating in general, are abundant. These limitations can include cost, convenience, food availability, attitude, concern for health, social support, knowledge, marketing and media, sociocultural acceptability, and physiology [10,11,12]. The public may recognize the individual messages (e.g., “consume less sodium”, “eat more fiber”, etc.), but not know how to implement the changes correctly in their diet on a daily basis [13]. Confusion exists over what makes foods healthy choices [14]. Social media trends typically do not follow DGA recommendations, but are readily accepted by consumers [15,16]. The NRF approach is positive messaging that can help consumers identify the best nutrition for their food dollars [4,8,17]. 

Due to chronic diseases typically developing over years, many people fail to recognize the risk associated with a nutrient poor diet until disease diagnosis, thereby lowering their interest in making changes when they would be most effective [3]. Recognition of risk is a strong motivator of behavior change [13]. Compared to their actual risk, Americans perceive themselves to be in less danger of developing cardiovascular disease [18] and type 2 diabetes [19]. Individuals viewed heart disease as more severe than other conditions, but perceived it also as controllable. In contrast, type 2 diabetes was seen as a less critical disease with lower risk of death when compared to other chronic diseases like cancer [20]. 

A major premise of the Health Belief Model (HBM) theory of behavior change is that the perception of one’s disease risk is a motivator to pursue health prevention actions. The HBM includes constructs of susceptibility, severity, benefits, barriers, and cues to action, which inter-relate to influence health behavior. It is important to recognize that the model centers on the individual’s strength of belief in these constructs and hazards, which may not agree with a professional evaluation of disease risk [21]. Applying the HBM in predictive models can identify which thematic constructs are associated with current behaviors. The HBM has been used as a tool to categorize questions and address the strength of each construct in individuals and/or successful behavior change interventions [21]. For example, cues to action and perceived barriers were shown to significantly affect intention to consume functional foods among older adults [22]. Perceived benefits and barriers significantly predicted willingness to use functional breads [23]. Self-efficacy, perceived benefits, and perceived barriers were significant predictors of willingness to choose organic foods [24]. 

The purpose of this study was to describe knowledge of NRFs, determine the presence of nutrition-related chronic disease conditions or risk factors, and assess the association of HBM constructs with the likelihood of eating NRFs by nutrition-related disease status. It was hypothesized that: (1) greater knowledge of NRFs will be predictive of increased likelihood of eating them, (2) persons with a nutrition-related disease or risk will have greater likelihood of eating NRFs, and (3) the HBM would be predictive of the likelihood of eating NRFs.

## 2. Materials and Methods

### 2.1. Study Design and Sample Recruitment

A national sample of 1300 adults, aged 18+, balanced for the U.S. Census age distributions of 18–44 and 45+, and equal gender representation was requested through the Survey Monkey^®^ Audience online panel service in November 2023. Survey Monkey (San Mateo, CA, USA) provided panelist data for U.S. geographic region. Two screener questions selected out those who did not live in the contiguous U.S. or were older than 80. Respondents who were missing key variables for analysis (112), had illogical answers (106), or failed two integrity check questions (44) were excluded. Sixty-two cases were removed from analysis for ‘speeding’, or completing the survey in less than 30% of the median completion time (10.0 min × 30% = 3 min) [25]. The final sample for analysis was 976. Completion of the survey was considered informed consent (IRB #22-353). 

#### 2.1.1. Survey Development

The online survey included 6 sociodemographic, 4 food choice, and 3 health status questions. The sociodemographic variables were age, gender, marital status, education level, household size [26], and a combined race and ethnicity question [27]. For food choice drivers, respondents indicated their role in food shopping [28]. The levels of importance of five food attributes (healthfulness, taste, price, convenience, and familiarity) were each ranked on a 5-point scale (not at all important = 1; extremely important = 5) [29]. Concern about food nutritional quality was asked with four response options (not at all concerned = 1; very concerned = 4) [30]. For health status, diet quality and personal health were ranked on a 5-point scale (poor = 1, excellent = 5) [28]. Participants reported the presence of one or more nutrition-related symptoms or chronic disease diagnosed by a medical professional (high blood pressure, high cholesterol, heart disease, type 2 diabetes, gastrointestinal disorder, or ‘other’) [28]. Two question sets on the knowledge of nutrient-rich foods, and the HBM constructs are described separately.

#### 2.1.2. Assessment of Knowledge of Nutrient-Rich Foods 

Participants were asked if they had previously heard of the term ‘nutrient-rich’ or ‘nutrient-dense’ foods (yes = 1, no = 0). After answering, the following definition of NRF suitable for laypersons was provided [9]. “The term ‘nutrient-dense’ indicates that there are more beneficial nutrients in a food (e.g., vitamins, minerals, lean protein, healthy fats, complex carbohydrates) compared to nutrients to limit (e.g., saturated fat, sodium, added sugars, and refined carbohydrates)” [9]. From a list of eight commonly consumed food choices, participants indicated which ones they believed were nutrient-rich. The four nutrient-rich food options of sweet potatoes, pinto beans, black beans, and carrots were selected from a nutrient-cost analysis, in which these foods were estimated to have 10% of the Daily Value for potassium and fiber within a serving size [8]. The nutrient-rich foods were then further confirmed with the use of the Nutrient Rich Foods Index [4]. USDA’s FoodData Central supported that the bean types were ‘good’ sources of iron, folate, and magnesium [31]. Carrots were considered a ‘good’ source of folate and Vitamin A. Sweet potatoes were confirmed to be a ‘good’ source of Vitamin C and Vitamin A [31]. The non-nutrient-rich food options (apple juice, doughnuts, iceberg lettuce, bacon) were chosen due to their increased concentration of saturated fat, added sugars, or low fiber content per serving size, [4,8,32]. One point was given for the correct classification of each item to form a nutrient-rich knowledge score ranging from 0 to 8). 

#### 2.1.3. Health Belief Model (HBM) Constructs 

The outcome variable of ‘likelihood of eating NRFs’ and the HBM construct scales were derived from theory, principal components factor loadings, and reliability testing. Unless otherwise noted, these perception or attitudinal questions had 5-point Likert scale options with varying response categories. The Cronbach’s alpha values for reliability, descriptive statistics for individual items, and the resulting HBM scales are shown in Table 1. 

Intent, or ‘likelihood of eating NRFs’, was comprised of Likert-type questions on participants’ encouragement of others to eat NRFs, reciprocal encouragement by social networks to eat NRFs [33], healthfulness as an important driver of food choice [29,34], and concern about the nutritional content of foods [30]. 

The specific question details and sources for the HBM constructs are described below. Perceived Benefits was represented by one question on the beneficial effects of eating NRF [33]. It was measured with a 5-point Likert scale (strongly disagree = 1, strongly agree = 5). 

The Perceived Barriers construct was estimated by five Likert attitude statements on reluctance to change one’s diet and disbelief that diet influences health [33]. Like the Perceived Benefits question, these statements were adapted from a national survey on fruit and vegetable attitudes and behaviors with the same 5-point Likert response categories [33].

Perceived Susceptibility for chronic disease risk was operationalized as the likelihood of experiencing chronic diseases (e.g., heart disease or stroke) on a 5-point Likert scale (much below average = 1, much above average = 5) [35]. 

Perceived Severity was defined as an awareness of diet-health consequences [21]. Respondents answered seven nutrition knowledge questions that focused on nutrition elements relevant to NRFs (dietary fat, fiber, calories, salt, micronutrients) and their links to chronic disease conditions [36]. Correct answers (0/1) were summed to create a score ranging from 0 (low knowledge) to 7 (high knowledge) [21]. 

Cues to Action was measured with three statements asking if participants would change behavior if recommended by medical professionals, the mass media, and friends or family [37]. Self-efficacy to consume a nutritious diet was measured by the summation of two statements on a 5-point Likert scale (strongly disagree = 1, strongly agree = 5) reflecting a two-week period [37].

### 2.2. Data Analysis and Transformations

All statistical analyses and data transformations were conducted using SPSS Statistics and SPSS Amos software (version 26.0, Chicago, IL, USA: IBM SPSS). Comparisons of categorical variables by the presence of a nutrition-related disease and gender were evaluated using Pearson chi-square tests. Multivariate scales for HBM constructs were all normally distributed. Bivariate demographic variables used within regression and modeling were coded as follows: gender (0 = man, 1 = woman), children in the household (0 = no children, 1 = 1 + children), marital status (0 = single, divorced, widowed, 1 = married or partnered). Only variables showing significant relationships with the outcome variable were utilized for the structural equation model to reduce diagrammatic and interpretative complexity. All *p*-values equal to or less than 0.050 were considered significant.

## 3. Results

The national survey sample approximated 2022 U.S. Census distributions for age categories 18–25 (6.6% vs. 8.7% nationally), 26–34 (12.9% vs. 12.3%), and 55–64 (9.6% vs. 12.9%) [38]. There were more sample respondents in the 35–54 category (38.7% vs. 25.7%) and slightly fewer proportionally in the 65+ group (9.1% vs. 17.4%) than for the U.S. overall. For race and ethnicity, the majority of respondents were non-Hispanic White (73% vs. 58.4% nationally), with smaller portions of Hispanics or Latinos (9.7% vs. 19.5%), African Americans (5.4% vs. 13.7%), Asians (5.4% vs. 6.4%) than in the general U.S. [38]. The regional distribution of responses vs. U.S. geographic region were higher for the Northeast (27.4% vs. 17.1%), lower for the South (31.8% vs. 38.6%), and about the same for the Midwest (19.9% vs. 20.6%) and the West (20.9% vs. 23.6%) [39]. 

Demographic and health characteristics are presented in Table 2. Slightly more than half had at least one nutrition-related chronic disease or predisposing condition (hereafter referred to as nutrition-related disease). This majority was significantly older, more likely to be men, married, and have higher education than those without a nutrition-related disease. High blood pressure, high cholesterol, and type 2 diabetes were the most frequently reported conditions. 

### 3.1. Assessment of Knowledge of Nutrient-Rich Foods

Table 3 shows the correct classification of eight food items by nutrition-related disease status. Significantly more people with a nutrition-related disease misclassified apple juice, bacon, and doughnuts as NRFs compared to those without a nutrition-related disease (all *p* ≤ 0.001). The NRF knowledge summary scale mean was significantly higher for those without a nutrition-related disease (5.9 vs. 5.6 out of 8). 

### 3.2. Health Belief Model

Table 1 shows the individual questions within each HBM construct and the total ranges, means, and standard deviations of the scales and the Perceived Severity score, as well as the means and standard deviations for individual components. The Cronbach alpha values for the five composite variable scales ranged from 0.695 to 0.887, indicating acceptable to good reliability [40]. Perceived Severity does not have an alpha value because it is a summary score and not a scale. Four of the six HBM construct means were significantly higher for those with a nutrition-related disease (perceived benefits, perceived barriers, perceived susceptibility or risk, and cues to action; (all *p* ≤ 0.001). The mean outcome variable ‘likelihood of eating NRFs’ scale was also significantly higher for those participants with a nutrition-related chronic disease (*p* < 0.001) 

A general linear model (GLM) was used to test if sociodemographic predictors (age, gender, race, education, main food shopper, children in the household, marital status, NRF knowledge, presence of nutrition-related disease condition) and the six HBM constructs explained variations in values of the outcome variable of the ‘likelihood of eating NRFs.’ The most parsimonious model, which excluded race, perceived barriers, and perceived severity, had adjusted *R*^2^ of 0.435. Specific analysis details are shown in Table 4. The model included demographic variables age (*p* = 0.033), gender (*p* = 0.057), education (*p* < 0.001), main food shopper (*p* < 0.001), presence of children in the household (*p* < 0.001), NRF knowledge (*p* < 0.003), presence of a nutrition-related disease (*p* = 0.004), and marital status (*p* = 0.008). The GLM contained the HBM constructs of perceived benefits, cues to action, and self-efficacy (all *p* < 0.001), as well as perceived susceptibility, which was not significant (*p* = 0.129). Positive standardized regression coefficients (beta values) were observed for age (B = 0.011), education (B = 0.260), main food shopper (B = 0.704), the presence of children in the household (B = 0.729), nutrition-related chronic disease (B = 0.434), marital status (B = 0.435), perceived benefits (B = 0.366), cues to action (B = 0.347), and self-efficacy (B = 0.462). Negative beta values were observed with higher NRF knowledge (B = −0.113). 

**Figure 1:**
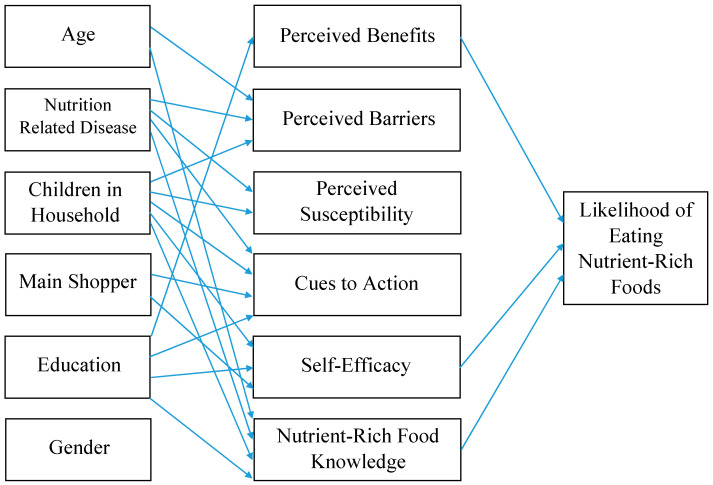
Structural Equation Model with Demographic and Health Belief Model Variables.

To provide a more fully elaborated analysis of interrelationships among these variables, a structural equation model (SEM) was estimated based on the HBM (Table 5, Figure 1), including age, gender, education, nutrition-related disease, presence of children in the household, main food shopper, NRF knowledge, perceived benefits, perceived barriers, perceived susceptibility, cues to action, and self-efficacy, all predicting the likelihood of eating NRFs. Through estimating simultaneous regression equations SEM incorporates the direct effects of each predictor variable on all outcomes, rather than estimating equations for each outcome separately. Thereby SEM takes into consideration complex patterns of how variables are related both within and across equations. 

Perceived severity and marital status did not contribute to the initial SEM and were excluded. Gender was retained due to its theoretical importance in the final SEM analysis although it was not significant. All other relationships were significant (*p* < 0.05). The model had a Minimum Discrepancy Function Divided by Degrees of Freedom (CMIN/DF) value of 1.648, which is a strong indication of good fit, as a value of 3 or less is indicative of a strongly fitting model [23]. The model had a Normed Fit Index (NFI) value of 0.977, in which a value of 0.9 or greater indicates a better-fitting saturated model. The SEM had a Comparative Fit Index (CFI) value of 0.991, in which a value closer to 1 indicates a better fit. The Root Mean Squared Error of Approximation (RMSEA) of 0.026, which is less than the benchmark of 0.05, indicating that the model fits the data closely given the degrees of freedom available in the model. The model had an Akaike Information Criterion (AIC) value of 198.503 compared to the independence AIC value of 1995.161. This test result suggests a better goodness of fit when the predictor variables are included in the model, in comparison to their ability to predict the likelihood of eating NRFs without the structured model [41]. 

As shown in Figure 1, demographic variables except for gender and main shopper were predictive of NRF knowledge in the SEM. Table 5 illustrates the maximum likelihood estimates for regression weights for the model variables. Higher education had a positive influence on perceived benefits. Positive influences on self-efficacy included having children, higher education, and being the main shopper of the household. Lastly, NRF knowledge was influenced positively by age and education, yet negatively by nutrition-related disease and children in the household. HBM variables of self-efficacy (Estimate 0.605), perceived benefits (Estimate 0.515), and NRF knowledge (Estimate −0.162) were significant associated with the likelihood of eating NRFs.

## 4. Discussion

This study investigated consumer knowledge of NRFs and whether the HBM constructs are related to the likelihood of eating these types of foods in a nationally representative sample. Hypothesis (1) that increased knowledge of NRFs would predict the likelihood of eating NRFs was not supported by our findings. Rather, those with higher knowledge of NRFs were less likely to eat them. Previous studies that investigated the effect of knowledge on dietary intake have shown findings ranging from low to high correlations [42,43]. A similar study with a nationwide sample of adults in the U.S. found nutrition knowledge did not have a strong effect on diet quality and suggested social, cultural, and political factors may outweigh the effects of nutrition knowledge and may be a more appropriate focus for dietary intervention and research [21]. Among low-income U.S. women, researchers found participants were well aware of nutritional recommendations, but family dynamics and socioeconomic pressures influenced food purchases more so than knowledge [12]. Our findings concur with this study, as the ability to identify NRFs did not improve the likelihood to consume them. However, increasing cues to action through social support of family members or friends might provide channels to facilitate use of NRFs particularly for the influence of family members or friends on food choice. Thus, social support and pressures may have a large impact on the likelihood to eat NRFs.

These survey data do support hypothesis (2) that the presence of a nutrition-related disease would increase the likelihood of eating NRFs. The positive association suggests chronic disease may be a catalyst in modifying one’s intentions for consuming NRFs. Having a nutrition-related disease has a positive association with perceived barriers, susceptibility, and cues to action in the GLM. Although those with a nutrition-related disease in our study had lower NRF knowledge scores and no difference in perceived severity (diet-health consequences) scores, a Polish study found adults with chronic diseases to have a higher level of awareness of diet-related diseases and dietary risk factors [44]. This information suggests that the driving forces behind improved behavior of an individual with chronic disease may be cues to action and a conscious realization of increased susceptibility to health conditions. However, the individuals in our sample had higher perceived barriers scores which suggests future programming and education should focus on addressing some of the components in this HBM construct such as willingness and ability to try new foods.

Hypothesis (3) that the HBM is a strong predictor of the likelihood of eating NRFs was supported. The SEM reinforced the finding that self-efficacy and perceived benefits were influential on the likelihood to eat NRFs. These results are consistent with other studies showing self-efficacy and perceived benefits as significant predictors on the willingness to eat organic foods [24] and perceived benefits as a significant HBM construct in a calcium education intervention [45]. The latter study also found significance with perceived susceptibility, severity, and barriers on the ability to increase dietary calcium intake. However, these three HBM constructs were not significant in our sample.

Self-efficacy (which includes principal sources of performance mastery, social modeling, social persuasion, and physiological states) was found to be a key component to support healthy behaviors and provide effective self-management of chronic disease in other research [46]. Social support by friends, family, and dietitians in addition to health literacy reinforced adherence to diet for those with type 2 diabetes [47].

Results suggest the HBM is highly applicable in predicting the likelihood of nutrition behavior, with self-efficacy having the greatest influence on the likelihood to consume NRFs. We concluded that more work should be done in the effort to educate and counsel individuals with a nutrition-related chronic disease on areas to support self-efficacy, social support, and awareness of the perceived benefits of NRFs.

## 5. Limitations

There are several limitations to this study. All data were derived from self-reports from anonymous national survey panel participants. Although two integrity checks were included, and data carefully screened, respondent bias may be present. The information collected is reliant upon proper recall and interpretation of questions. As a cross-sectional survey, significant associations between variables do not indicate causality. Time since diagnosis, severity of disease or risk factor(s), health literacy, previous nutrition or health education, availability of healthcare, and income data were not collected. Such measures may give a broader view of other socioeconomic drivers of food knowledge, choice, and health. This survey measures perceptions of HBM constructs without verifiable measurements of NRF or other dietary intake behaviors.

## 6. Conclusions

For health professionals and nutrition educators, these findings offer valuable insights for predicting the likelihood of eating NRFs among U.S. adults. The HBM can be implemented throughout the education and counseling process and targeted interventions. The ability of consumers to identify NRFs has applications in both future research and current practice. Our findings indicate lower knowledge of NRFs for those with a nutrition-related disease. Healthcare professionals should highlight NRFs within nutrition education and messaging as a means to maximize healthy food choices. Future research would benefit from analysis of the relationship of consumer knowledge with NRFs and which NRF foods are most identifiable by consumers.

This study did not demonstrate conclusively that individuals with higher NRF knowledge have a greater likelihood of eating these foods. Further research is needed to explain the meaning of this phenomenon and ultimately evaluate the success of current nutrition education models within this sector.

However, our study supports that individuals with a nutrition-related disease have a higher likelihood of eating NRFs, and the HBM is a valuable tool in nutrition research. Respondents with a nutrition-related disease did have greater perceived barriers and cues to action scores. Therefore, when working with these individuals an emphasis on reducing perceived barriers and social pressures should be promoted. Of the constructs in this model, self-efficacy had the greatest influence, suggesting strengthening empowerment and identifying ways to promote it within nutrition counseling may yield the greatest change. Nutrition professionals should discuss barriers and motivators to food choices beyond knowledge with clients. Lastly, nutrition knowledge of NRFs proved not to be positively associated with the likelihood of eating NRFs, suggesting nutrition education should go beyond just the identification of NRFs, and focus on the benefits of consuming them.

## Figures and Tables

**Table 1:** Health Belief Model Construct composition (*n* = 976).

Outcome Variable	Mean ± SD
Likelihood of Eating Nutrient-Rich Foods Scale (Range 0–19)	13.82 ± 2.77
Healthfulness as an important driver of food choice	3.92 ± 0.97
I often encourage my family and friends to eat nutrient-rich foods	3.45 ± 1.08
My family and friends often eat nutrient-rich foods when we are together	3.28 ± 1.01
Concern about nutritional content in food	3.28 ± 0.73
Cronbach’s alpha = 0.723	
Perceived Benefits:	
It is beneficial to eat nutrient-rich foods	4.32 ± 0.79
Perceived Barriers Scale (Range 0–25):	14.42 ± 4.01
When it comes to food, I’m a creature of habit	3.57 ± 1.03
Dinner doesn’t seem right without meat	3.39 ± 1.22
It is hard for me to eat nutrient-rich foods because I don’t know what they are	2.99 ± 1.14
I do not like the taste of beans	2.36 ± 1.31
What I eat does not really affect my health	2.11 ± 1.17
Cronbach’s alpha = 0.712	
Self-Efficacy (over the next two-week period…) Scale (Range 0–10)	7.84 ± 1.68
If I tried, I am confident that I could maintain a diet high in nutrition most of the time	3.91 ± 0.89
If I wanted to, I feel I would be able to follow a diet high in nutrition most of the time	3.93 ± 0.89
Cronbach’s alpha = 0.887	
Perceived Severity (diet-health consequences) Score (0/1); (Range 0–7)	3.63 ± 1.64
Eating less salt protects against heart disease	0.79 ± 0.41
Fiber protects against heart disease	0.65 ± 0.48
Red meat does not protect against heart disease	0.60 ± 0.49
Saturated fat raises cholesterol	0.46 ± 0.50
Not eating fruits and vegetables is a cause of chronic disease	0.45 ± 0.50
Folic acid is linked to neural tube defects	0.39 ± 0.49
Fat has more calories per gram	0.29 ± 0.45
Perceived Susceptibility or Risk to Chronic Disease Scale (Range 0–20)	11.70 ± 3.50
Likelihood of experiencing high blood pressure	3.03 ± 1.07
Likelihood of experiencing heart disease or stroke	2.93 ± 1.06
Likelihood of experiencing diabetes	2.89 ± 1.14
Likelihood of experiencing cancer	2.85 ± 1.03
Cronbach’s alpha = 0.827	
Cues to Action to Change Food Choices Scale (Range 0–15)	10.92 ± 2.24
I would pay more attention to the quality of my food choices … … if recommended by a doctor or medical professional	4.08 ± 0.83
… if my friends or family members mentioned it	3.55 ± 0.92
… if I read information in the mass media	3.30 ± 1.07
Cronbach’s alpha = 0.695	

**Table 2:** Demographic characteristics of U.S. adults aged 18–80 by presence of a nutrition-related disease condition (*n* = 976).

	Total	No Nutrition-Related Disease 45.3% (*n* = 442)	Has Nutrition-Related Disease 54.7% (*n* = 534)	*p*-Value
Age in years ($\bar{x}$ ± SD)	44.8 ± 14.3	40.2 ± 13.6	48.6 ± 13.8	*p* < 0.001
	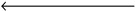 % 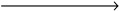			
Gender Man Woman	48.3 51.7	43.4 _a_ 56.6 _a_	52.2 _b_ 47.8 _b_	*p* = 0.006
Presence of nutrition-related disease (could report more than 1) High blood pressure High cholesterol Type 2 diabetes Gastrointestinal disorder Heart disease	28.8 25.9 17.3 14.4 6.9	N/A	52.6 47.4 31.6 26.4 12.5	
Marital Status Single/Divorced/Widowed Married/Living with partner	29.9 70.1	34.2 _a_ 65.8 _a_	26.4 _b_ 73.6 _b_	*p* = 0.008
Children in household No children One child+ in household	49.7 50.3	52.0 48.0	47.8 52.2	n.s.
Years of Education 9–12th grade and/or GED Some college, no degree Associate degree, Tech school Bachelor degree Masters, Doctoral, Professional degree	14.1 13.8 13.9 32.1 26.0	16.3 _a_ 12.9 _a_ 14.0 _a_ 38.0 _a_ 18.8 _a_	12.4 _a_ 14.6 _a_ 13.9 _a_ 27.2 _b_ 32.0 _b_	*p* < 0.001
Race/Ethnicity White Other	76.9 23.1	74.4 25.6	79.0 21.0	n.s.

Same subscript letters indicate column proportions that are not significantly different from each other.

**Table 3:** Percentage of U.S. adults aged 18–80 who correctly identified nutrient-rich foods (NRFs) by presence of a nutrition-related disease condition (*n* = 976).

Food Item	Total	No Nutrition-Related Disease 45.3% (*n* = 442)	Has Nutrition-Related Disease 54.7% (*n* = 534)	*p*-Value
	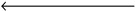 % 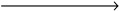			
Heard of Nutrient-Rich Foods Term Yes No	84.4 15.6	83.9 16.1	84.8 15.2	*n.s.*
Not Nutrient-Rich				
1. Doughnuts Correct Incorrect	86.5 13.5	91.6 _a_ 8.4 _a_	82.2 _b_ 17.8 _b_	*p* < 0.001
2. Bacon Correct Incorrect	85.6 14.4	89.6 _a_ 10.4 _a_	82.2 _b_ 17.8 _b_	*p* = 0.001
3. Apple Juice Correct Incorrect	82.0 18.0	89.1 _a_ 10.9 _a_	76.0 _b_ 24.0 _b_	*p* < 0.001
4. Iceberg Lettuce Correct Incorrect	79.6 20.4	80.8 19.2	78.7 21.3	*n.s.*
Nutrient-Rich				
5. Black Beans Correct Incorrect	64.5 35.5	64.5 35.5	64.6 35.4	*n.s.*
6. Sweet Potato Correct Incorrect	62.8 37.2	62.7 37.3	62.9 37.1	*n.s.*
7. Pinto Beans Correct Incorrect	58.9 41.1	59.0 41.0	58.8 41.2	*n.s.*
8. Carrots Correct Incorrect	54.4 45.6	55.9 44.1	53.2 46.8	*n.s.*
Nutrient-Rich Foods Knowledge Score (sum of items 1–8; $\bar{x}$ ± SD)	5.74 ± 1.8	5.93 ± 1.7	5.59 ± 1.9	*p* = 0.003

Same subscript letters indicate column proportions that are not significantly different from each other. Non-significant *p* values are indicated by n.s.

**Table 4:** Parameter Estimates of a General Linear Model: Measuring the Strength of Health Belief Model Constructs in Predicting the Likelihood to Consume Nutrient Rich Foods (NRF) (*n* = 976) *.

	Beta (*p*-Value)	Partial Eta Squared	Observed Power
Demographic Variables			
Age	0.011 (0.033)	0.005	0.570
Gender (Woman = 1)	0.263 (0.057)	0.004	0.477
Education	0.260 (0.000)	0.025	0.999
Main Shopper (Yes = 1)	0.704 (<0.001)	0.019	0.992
Children in the Household (Yes = 1)	0.729 (<0.001)	0.021	0.995
Nutrient-Rich Foods Knowledge	−0.113 (0.003)	0.009	0.839
Nutrition-Related Disease Condition (Yes = 1)	0.434 (0.004)	0.009	0.828
Marital Status (Married = 1)	0.435 (0.008)	0.007	0.761
Health Belief Model Constructs			
Perceived Benefits	0.366 (<0.001)	0.015	0.970
Cues to Action	0.347 (<0.001)	0.091	>0.999
Self-Efficacy	0.462 (<0.001)	0.094	>0.999
Perceived Susceptibility	−0.030 (0.129)	0.002	0.329
Model Intercept	5.419 (<0.001)	0.074	>0.999

* Model Adj *R*^2^ = 0.435, F value = 63.625, Partial Eta Squared = 0.442, Observed Power > 0.999, and model *p*-value < 0.001.

**Table 5:** Maximum Likelihood Estimates for Regression Weights in the Structural Equation Model: Assessing the Applicability of the HBM Constructs to Predict the Likelihood to Eat Nutrient-Rich Foods (NRF) (*n* = 976; → signifies influence).

Independent Variable	Dependent Variable	Estimate	S.E.	C.R.	*p*-Value
Age	Perceived Barriers	−0.024	0.009	−2.745	*p* = 0.006
Age	NRF Knowledge	0.010	0.004	2.212	*p* = 0.027
Nutrition Related Disease	Perceived Barriers	1.258	0.254	4.948	*p* < 0.001
Nutrition Related Disease	Perceived Susceptibility	1.720	0.217	7.928	*p* < 0.001
Nutrition Related Disease	Cues to Action	0.616	0.126	4.906	*p* < 0.001
Nutrition Related Disease	NRF Knowledge	−0.403	0.120	−3.361	*p* < 0.001
Children in Household	Perceived Barriers	1.832	0.256	7.164	*p* < 0.001
Children in Household	Perceived Susceptibility	0.582	0.217	2.684	*p* = 0.007
Children in Household	Cues to Action	0.963	0.132	7.303	*p* < 0.001
Children in Household	Self-Efficacy	0.271	0.099	2.745	*p* = 0.006
Children in Household	NRF Knowledge	−0.702	0.121	−5.779	*p* < 0.001
Main Shopper	Cues to Action	0.457	0.146	3.128	*p* = 0.002
Main Shopper	Self-Efficacy	0.319	0.113	2.832	*p* = 0.005
Education	Perceived Benefits	0.101	0.018	5.634	*p* < 0.001
Education	Self-Efficacy	0.239	0.038	6.240	*p* < 0.001
Education	Cues to Action	0.260	0.048	5.384	*p* < 0.001
Education	NRF Knowledge	0.127	0.040	3.147	*p* = 0.002
Self-Efficacy	Likelihood to eat NRFs	0.605	0.046	13.156	*p* < 0.001
Perceived Benefits	Likelihood to eat NRFs	0.515	0.098	5.253	*p* < 0.001
NRF Knowledge	Likelihood to eat NRFs	−0.162	0.040	−4.082	*p* < 0.001

## Data Availability

The data presented in this study are available upon request from the corresponding author.
